# Increased diversity of *Malassezia* species on the skin of Parkinson’s disease patients

**DOI:** 10.3389/fnagi.2023.1268751

**Published:** 2023-10-03

**Authors:** Xinyu Han, Janis Bedarf, Sabine Proske-Schmitz, Ina Schmitt, Ullrich Wüllner

**Affiliations:** ^1^Department of Neurology, University of Bonn, Bonn, Germany; ^2^DZNE, German Center for Neurodegenerative Diseases, Bonn, Germany

**Keywords:** Parkinson’s disease, *Malassezia*, odor, sebum, biomarker

## Abstract

**Background:**

Parkinson’s disease (PD) is characterized by motor disorders and the composition of Lewy bodies (LBs) in the substantia nigra. Due to the lack of a definitive biomarker, the current treatments do not modify the progression of PD. Recently, researchers revealed lipid dysregulation and some potential volatile biomarkers of PD related to a unique odor from PD patients by metabolomics of sebum, which is supposed to cause a potential change for skin microflora. In this study, we identified the 4 *Malassezia* species in PD patients and compared them with healthy controls.

**Methods:**

We collected 95 sebum samples (47 PDs and 48 Controls) by cotton swabs and extracted the DNA. The identification of *Malassezia* species was performed by Nested PCR. Specific primers for each species were used to amplify corresponding yeasts in each sample.

**Results:**

*M. restricta* and *M. globosa* are the most common species for both groups. The prevalence of *M. slooffiae* and *M. sympodialis* were significantly higher in the PD group compared with controls (63.8% vs. 29.1 and 74.5% vs. 54.2% respectively), the binary logistic regression model further indicated that *M. slooffiae* (OR = 9.358, *p* < 0.001) was associated with PD. Moreover, the diversity of *Malassezia* species was significantly greater (3.5 vs. 2.9 species per individual, *p* = 0.002) in the PD group.

**Conclusion:**

Based on our results, we preliminarily observed a change in *Malassezia* species incidence and diversity on the skin of PD patients, which could be associated with lipid dysregulation; meanwhile, it might also be a noninvasive biomarker for PD.

## Introduction

Parkinson’s disease (PD) is one of the most common progressive neurodegenerative disorders which is characterized by inducing motor disorders, rigidity, and tremor through the loss of dopaminergic neurons in the substantia nigra (Pisa et al., 2020; Niemann et al., 2021). PD affects more than 6 million people worldwide, of which the prevalence of patients over 65 years of age is about 1.8%, and as the aging demographics, the overall prevalence is expected to increase (Laurence et al., 2019; Pisa et al., 2020). Although it is currently known that the main pathological hallmark of PD is the formation of Lewy bodies, the etiology of PD still has not been fully understood.

In addition to the motor symptoms, non-motor symptoms and alterations of the autonomic nervous system are common in PD and may precede the diagnosis of PD. As early as 1927, Krestin reported that seborrheic facies as a cutaneous manifestation in PD patients (Ravn et al., 2017). Changes in the skin are common symptoms of PD patients; the skin of many patients is shiny, greasy, or flaky, especially on the face and scalp. Interestingly, Morgan reported that Joy Milne, who is a human super smeller, smelled a unique, musky odor on the skin of her husband with PD (Morgan, 2016). In the following pilot study, she successfully identified 11 out of 12 PD patients or control by smelling, except one misidentified control subject who also had the special odor; surprisingly, this control subject was later diagnosed with PD (Morgan, 2016). Barran et al. have indicated that the source of the odor was associated with altered biochemical composition of the sebum, and localized to the back of the neck (Trivedi et al., 2019). There are many sebaceous glands in this region of skin that produce sebum. We hypothesized that changes in the skin microflora of PD patients might be involved as well, such as *Malaasezia*.

*Malassezia*, lipophilic fungi, is the most prevalent member of cutaneous microbiota in normal humans and other animals (Laurence et al., 2019; Malinovská et al., 2019). The *Malassezia* yeasts currently include 17 species, the most frequently found species in humans include *M. restricta*, *M. globosa*, *M. slooffiae*, and *M. sympodialis* (Arendrup et al., 2014; Malinovská et al., 2019). Others, i.e., *M. dermatis* and *M. japonica*, occur in higher frequencies in East Asia only, while some, such as *M. furfur* are predominant in neonates (Boekhout et al., 2010; Velegraki et al., 2015). In humans, *Malassezia* inhabits the skin in sebum-rich areas, such as the face and scalp; and the distribution of *Malassezia* species varies by body part and age but not by gender (Prohic, 2014). In addition, under certain conditions, *Malassezia* is also an opportunistic organism and cause various skin disease, such as *M. furfur* (Malinovská et al., 2019). For example, *Malassezia* plays an important role in the development of seborrheic dermatitis (SD) which is a common chronic inflammatory skin disease (Ravn et al., 2017).

In this study, the main purpose is to detect the potential change in the distribution of *Malassezia* species in PD patients and controls. Considering the relationship between *Malassezia* and skin diseases, we collected the sebum samples from the individuals without skin disease and identified the distribution of *Malassezia* species using nested PCR.

## Materials and methods

### Materials

A total of 95 sebum samples were collected by swabbing from 47 patients with PD and, 48 controls. The retroauricular skin was rubbed with a sterile cotton swab (HARTMANN, Germany), which was stored in a sterile 15 mL centrifuge tube at −80°C until DNA extraction. The clinical information of each participant is shown in Table 1. All PD patients were clinically diagnosed and treated in the Department of Neurology, University Hospital Bonn. Only PD patients without any overt, skin diseases, especially seborrheic dermatitis (SD), based on the clinical exam will be included. Meanwhile, the spouse control or age-matched healthy control individuals were also collected. The study received informed consent from all participants (ethics approval 145/17).

**Table 1:** Description of PD patients and controls, distribution of *Malassezia* species in PD patients and controls.

	Controls (*n* = 48)	PDs (*n* = 47)	OR (95% CI)^1^	*p* ^2^	*p* ^3^
Age	65 ± 16.2	71 ± 7.5		*p* = 0.218	
Sex (M/F)	21/27	35/12	8.592 (2.639–27.970)	*p* < 0.01	*p* < 0.001
*Malassezia* species diversity	2.9 ± 0.78	3.5 ± 0.83		*p* < 0.01	
*M. Restricta* (P/N)	48/0 (100%)	47/0 (100%)		*p* = 1.000	
*M. globosa* (P/N)	48/0 (100%)	47/0 (100%)		*p* = 1.000	
*M. Sloofiae* (P/N)	14/34 (29.1%)	30/17 (63.8%)	9.358 (2.931–29.880)	*p* < 0.001	*p* < 0.001
*M. Sympodialis* (P/N)	26/22 (54.2%)	35/12 (74.5%)		*p* < 0.05	*p* = 0.770

PD, Parkinson’s disease; M, male; F, female; P, positive; N, negative.

All ages are expressed in years.

^1^OR value: Female, M. Sloofiae negative and M. Sympodialis negative is the reference, respectively.

^2^*p*, obtained by the Chi-square test or the Wilcoxon rank sum test.

^3^*p*, obtained by the binary logistic regression model.

### DNA extraction and nested PCR

The DNA was extracted by a QIAamp DNA Mini Kit (QIAGEN N.V., Venlo, Netherlands) according to the manufacturer’s instructions; each DNA sample was eluted in 100ul AE buffer. Due to the high background DNA from human tissue, we chose a nested PCR approach to increase the specificity for *Malassezia*. The 1st PCR primers and species-specific primers for nested PCR were taken from the internal transcribed spacer region of the rRNA gene, according to Zhang, et al. (Hao et al., 2014).

The first amplification was performed in a 25ul PCR reaction system, the mixture consisted of 2 μL extracted DNA from each sample, 12.5ul 2× HotStarTaq Plus Master Mix (QIAGEN, Germany), 0.5 μL 10 μM F primer and R primer (F: 5’-ACCTGCAGAAGGAT CATTAGTGA-3′ and R: 5’-TCCTCCGCTTATTGATATGC-3′), add water to 25ul. PCR was conducted in a Thermal Cycler (Biometra TAdvanced, Analytik Jena AG, Germany) with a program consisting of an initial denaturation for 5 min at 95°C, followed by 25 cycles of 95°C for 30s, 52°C for 45 s, 72°C for 1 min, and a 7 min final extension at 72°C.

For the second amplification, one forward primer (*Malassezia* 2nd F: 5′- GTGAATTGCAGAATTCCGTGAAT −3′) was combined with one of the following species-specific reverse primers:

*M. restricta* R: 5’-GCGAGCCTGTGCTAGGTA-3′.

*M. globosa* R: 5’-GAGCTTTTTCTAGAGAAGAAAAG-3′.

*M. slooffiae* R: 5’-CTTTTCGAGCGAGCCTACCAA-3′.

*M. sympodialis* R: 5’-TACAATCCCCAGGCAGCAA-3’.

Specifically, 1 μL unpurified 1st PCR product was amplified in a 25ul reaction mixture, the mixture also included 12.5ul 2× HotStarTaq Plus Master Mix, 0.5 μL 10 μM F primer and R primer, 2.5ul Coraload, add water to 25ul. Thermocycling conditions consisted of an initial denaturation for 5 min at 95°C, followed by 25 cycles of 95°C for 30s, 58°C for 45 s, and 72°C for 45 s, and a 7 min final extension at 72°C. Positive and negative controls were included in each amplification. Sizes of the expected products were confirmed by agarose gel electrophoresis. The specificity of the different primer pairs was verified by sequencing (not shown).

### Production of positive controls and DNA sequencing

We applied PCR product cloning to synthesize the positive controls for each *Malassezia* species. The individual *Malassezia* species PCR fragments were purified from agarose gel by Gel DNA Recovery Kit (ZYMO RESEARCH, United States) and cloned in pJET1.2 using the CloneJET PCR Cloning Kit (Thermo Scientific, United States) according to the manufacturer’s instructions. Clones were sequenced (Eurofins Genomics) and inserted fragments verified by BLAST search.^1^ In addition, the specificity of each positive control was also confirmed by PCR.

### Statistical analysis

SPSS 21.0 statistical analysis software was used to analyze the experimental data. Age and *Malassezia* species diversity are presented as the mean ± standard deviation, categorical variables are presented as the percentage. The Wilcoxon rank sum test was applied to analyze the age and *Malassezia* species diversity. The chi-square test was applied to analyze the differences in the categorical variables including different *Malassezia* species and sex. The binary logistic regression model was performed to explore the potential *Malassezia* species related to PD. The diagnosis of PD served as a dependent variable; sex, *M. slooffiae*, and *M. sympodialis* as independent variables. Values were considered significant for *p* < 0.05.

## Results

### The specificity of species-specific primer pairs and positive controls

**Figure 1:**
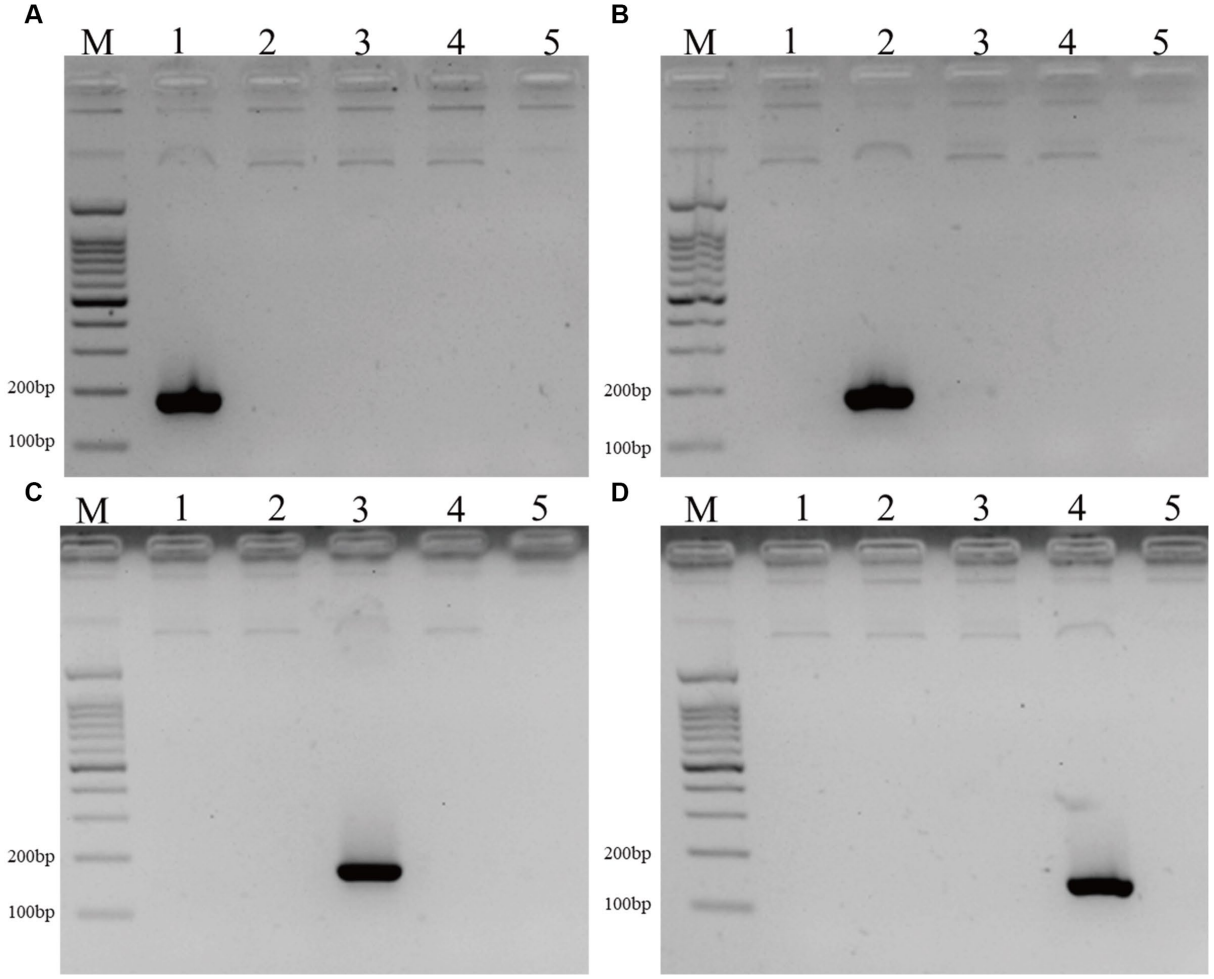
The DNA fragments of positive controls and 4 *Malassezia* species in PD patients and controls. **(A–D)** The positive controls of *M. Restricta* (183 bp, **A**), *M. globosa* (187 bp, **B**), *M. Sloofiae* (192 bp, **C**), and *M. Sympodialis* (171 bp, **D**) can only be amplified by the matched species-specific primer pairs (Lane M: 100 bp ladder; Lanes 1–5: *M. restricta*, *M. globosa*, *M. slooffiae*, *M. sympodialis*, negative control.

The specificity of different primer pairs was confirmed by sequencing, the four species-specific primer pairs are specific to the corresponding *Malassezia* species. Meanwhile, the four positive controls can only be amplified by the matched species-specific primer pairs (Figure 1).

### The analysis of clinical data and diversity of *Malassezia* species among individuals

We collected 95 sebum samples from 47 PD patients and 48 controls. There is no difference in age between the PD and control group, but there are more male participants in the PD group compared with controls (*p* < 0.05). The diversity of *Malassezia* species was significantly greater (3.5 vs. 2.9 species per individual, *p* < 0.05) in the PD group (Table 1).

### The incidence of different *Malassezia* species in PD patients and controls

**Figure 2:**
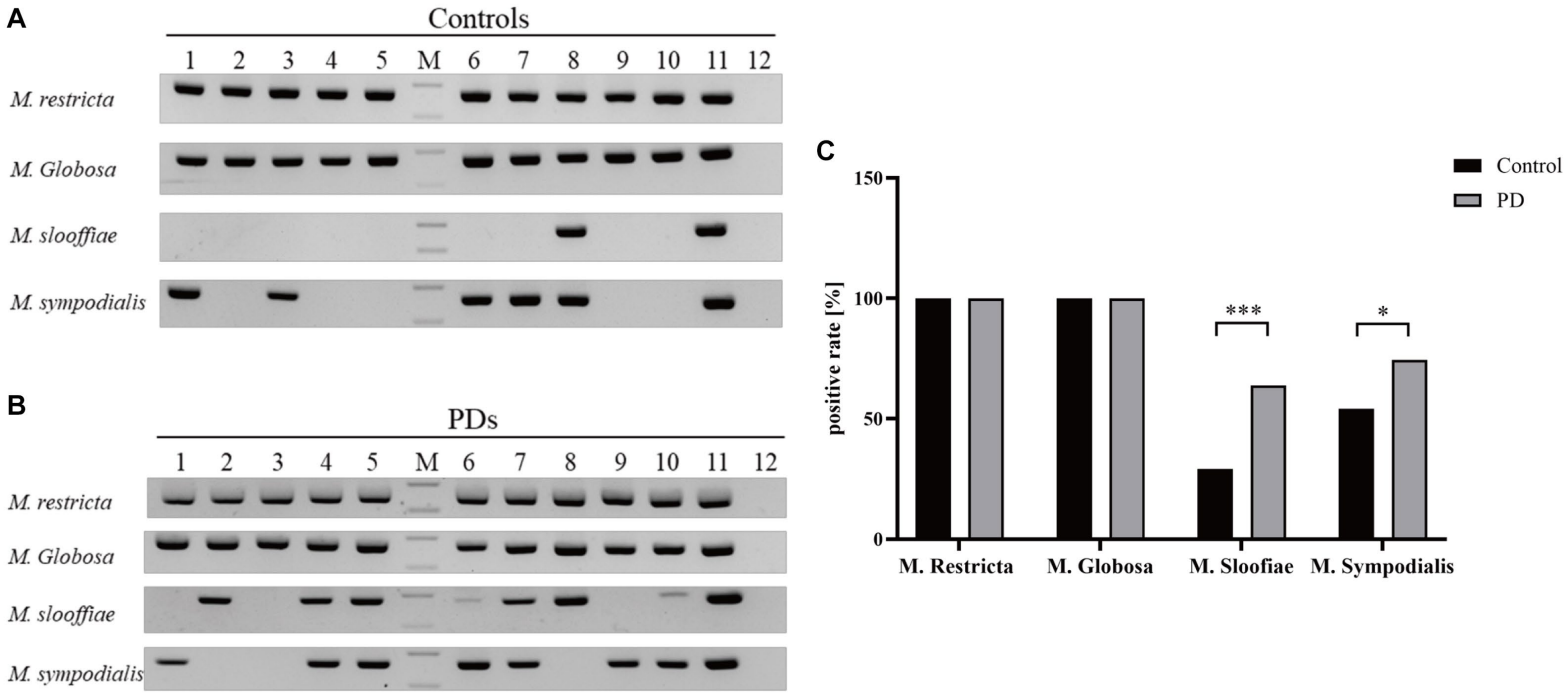
The positive rates and DNA fragments of four *Malassezia* species in PD patients and controls. **(A)** The DNA fragments of four *Malassezia* species in 10 controls (Lanes1–12: Controls1–10, positive control and negative control, M: DNA ladder, upper band 200 bp, lower band 100 bp). **(B)** The DNA fragments of four *Malassezia* species in 10 PD patients (Lanes1–12: PD patirents1–10, positive control and negative control, M: DNA ladder, upper band 200 bp, lower band 100 bp). **(C)** The positive rates of different *Malassezia* species (^***^p < 0.001; ^*^p < 0.05).

After the second amplification, there are specific bands for each *Malassezia* species. Representative results of 10 PD patients and controls are displayed in Figures 2A,B.

*M. restricta* and *M. globosa* are the most common two *Malassezia* species with a prevalence of 100% in PDs and controls (Figure 2C). Interestingly, the positive rates of *M. slooffiae* and *M. sympodialis* were 63.8 and 74.5% in PD patients respectively, which are significantly higher than in controls (*p* < 0.05) (Figure 2C). Meanwhile, the logistic regression model indicated that the high incidence of *M. slooffiae* (OR = 9.358, p < 0.05) was associated with PD, but not *M. sympodialis*. In addition, due to the different sex distribution between the two groups, we further analyzed the positive rate of *M. slooffiae* in males and females between PD and control groups, respectively. There is a similarly rising trend for its incidence in both sexes in PD compared with the control group (Table 2).

**Table 2:** The positive rate of *M. sloofiae* in different sexes between PD and control groups.

	Controls (*n* = 48)	PDs (*n* = 47)	*p*
Male (P/N)	2/19 (9.5%)	20/15 (57.1%)	*p* < 0.001
Female (P/N)	12/15 (44.4%)	10/2 (83.3%)	*p* < 0.05

PD, Parkinson’s disease; P, positive; N, negative; *p* value was obtained by Chi-square test.

## Discussion

To date, the diagnosis of PD mainly depends on the clinical motor symptoms; no cheap, reliable, and non-invasive biomarker is available to support its clinical diagnosis. Regrettably, almost half of the dopamine neurons are lost before motor symptoms emerge, thus a tool for early, pre-motor symptom diagnosis is urgently needed. In this study, we observed a change in *Malassezia* species diversity and incidence in PD patients based on a non-culture method, which could evolve into a potential non-invasive biomarker of PD.

The concept of skin as a mirror of PD pathology can be traced back to the early 20th century (Arsic Arsenijevic et al., 2014). Regarding the study in the last years, peripheral autonomic dysfunction may be an important source of cutaneous problems, such as impaired sweating and sebum production dysregulation (Dabby et al., 2006). The reduction of epidermal nerve fiber density and the loss of dermal nerve fiber in PD has been demonstrated before (Dabby et al., 2006; Navarro-Otano et al., 2015). Alpha-synuclein is the main protein component of Lewy bodies, its deposits in the central nervous system (CNS) have already been implicated in the pathogenesis of PD. Meanwhile, several studies repeatedly reported that misfolded alpha-synuclein deposit was also found in the peripheral autonomic nervous system, including skin biopsy (Donadio et al., 2014,^2016^; Doppler et al., 2014; Fayyad et al., 2019; Ma et al., 2019). Perhaps, alpha-synuclein deposits could contribute to the cutaneous autonomic dysfunction (Zange et al., 2015), which still needs to be further determined. Furthermore, some skin diseases such as Seborrheic dermatitis (SD) Bullous pemphigoid, and Melanoma are overrepresented in PD, which may be a long-term effect of autonomic dysfunction (Niemann et al., 2021). These emerging pieces of evidence indicated that the skin could also be a helpful tool in the diagnosis of PD.

*Malassezia*, as a lipid-dependent fungus, depends on host lipids for survival by secreting extracellular lipases (Laurence et al., 2019). Previously, most of the focus has been on *Malassezia* and related skin diseases such as SD. Interestingly, Tanner et al. found that the risk of PD is increased for individuals with a diagnosis of SD and SD might be a potential premotor feature of PD (Tanner et al., 2012); and Arsenijevic et al. reported that there is a positive correlation between SD, PD, and *M. globosa* presence and density (Arsic Arsenijevic et al., 2014). Seborrhea, defined as an elevated sebum secretion rate (SER), was also reported in the last century in PD patients (Laurence et al., 2019). Nevertheless, it’s notable that the relationship among SD, SER, and PD remains to be elucidated. Increasingly evidence suggests that SD and SER are completely different clinical entities; and SER in PD patients is also controversial, which is only found in some studies (Burton and Pye, 1983; Cowley et al., 1990; Laurence et al., 2019). Furthermore, several lines of evidence supported that *Malassezia* may directly contribute to PD: (1) many PD risk alleles affect the metabolism of lipids, (2) the invasiveness of *Malassezia* is stimulated by L-DOPA, and (3) low CD4 + T cell counts observed in PD might lead to the over-proliferation of *Malassezia* (Laurence et al., 2019). Sinclair et al. also revealed lipid dysregulation in PD patients’ skin by performing an LC–MS analysis of sebum (Sinclair et al., 2021). It could lead to a preference for particular Malassezia species (in PD patients), which may also account for the increased prevalence of SD in PD patients. Therefore, it is worth detecting whether PD will directly affect the presence and diversity of *Malassezia*. To our knowledge, this is the first study to detect the diversity of *Malassezia* species based on non-culture methods in PD patients without skin disease.

In our study, we observed that the predominant *Malassezia* species are *M. restricta* and *M. globosa* in PD patients and controls; the positive rate of *M. sloofiae* and *M. sympodialis* is significantly higher in the PD group compared to controls. According to the binary logistic regression model, we found a high incidence of *M. sloofiae* associated with PD. Moreover, the diversity of *Malassezia* species found in the PD group was significantly greater (3.5 species per individual) than in the group of controls (2.9 species per individual). As we mentioned above, dysregulated lipid metabolism and increased sebum secretion in PD patients’ skin likely underlie this phenomenon. Recently, Barran et al. discovered some potential volatile biomarkers of PD from sebum, and the smell of which was also described as a similar odor of PD patients by Joy; especially, eicosane and octadecanal, which are up-regulated in PD subjects (Trivedi et al., 2019). The elevated production of these highly lipophilic molecules may in part contribute to the prevalence of different Malassezia species since the specific exogenous lipids are required for its growth (Trivedi et al., 2019), which still needs to be further investigated. Therefore, our results in part proved Barran’s hypothesis that the change of these volatile substances and odor in PD patients suggests a change of skin microflora and skin physiology that is highly specific to PD.

Interestingly, although *Malassezia* is thought to be limited to the skin before, more and more studies reported finding *Malassezia* in internal organs, including in the central nervous system (CNS) of patients with certain neurodegenerative disorders. A recently published comparative study reported that the incidence of *Malassezia* in the CNS of multiple sclerosis is higher compared to the controls (Laurence et al., 2019). Another study found the presence of mixed microbes in the CNS of PD patients, including *Malassezia* (Pisa et al., 2020). Although these studies provide preliminary evidence that *Malassezia* may also be involved in the pathogenesis of PD and other neurodegenerative disorders, caution is warranted with regard to the presumed presence of microbes in brains.

Furthermore, there are still some limitations in our study, and further studies in a large asymptomatic cohort of the elderly are needed to determine their causal association and whether similar changes might precede the onset of motor symptoms. In addition, we are going to establish an approach to detect the prevalence of Malassezia and other cutaneous microbiota in PD by next-generation sequencing (NGS).

## Data availability statement

The raw data supporting the conclusions of this article will be made available by the authors, without undue reservation.

## Ethics statement

The studies involving humans were approved by the Clinical Ethics Committee, University Hospital Bonn. The studies were conducted in accordance with the local legislation and institutional requirements. Written informed consent for participation was not required from the participants or the participants’ legal guardians/next of kin because we just collect the sebum samples from the skin by swabs, which is non-invasive; at the same time, we obtained the verbal consent of the patients.

## Author contributions

XH: Conceptualization, Data curation, Investigation, Writing – original draft, Writing – review & editing, Methodology. JB: Conceptualization, Writing – review & editing. SP-S: Methodology, Writing – review & editing. IS: Writing – review & editing, Data curation. UW: Conceptualization, Project administration, Writing – review & editing.

## Footnotes

^1^ http://www.ncbi.nlm.nih.gov
